# Pathogenic effect of a *TGFBR1* mutation in a family with Loeys–Dietz syndrome

**DOI:** 10.1002/mgg3.943

**Published:** 2019-09-01

**Authors:** Luc Cozijnsen, Astrid S. Plomp, Jan G. Post, Gerard Pals, Natalija Bogunovic, Kak K. Yeung, Hans W. M. Niessen, Marie‐José T. H. Goumans, Daniela Q. C. M. Barge‐Schaapveld, Dimitra Micha

**Affiliations:** ^1^ Department of Cardiology Gelre Hospital Apeldoorn The Netherlands; ^2^ Department of Clinical Genetics Amsterdam University Medical Centre, AMC Amsterdam The Netherlands; ^3^ Department of Genetics University Medical Centre Utrecht The Netherlands; ^4^ Department of Clinical Genetics Amsterdam University Medical Centre, VUMC, Amsterdam Cardiovascular Sciences Amsterdam The Netherlands; ^5^ Department of Physiology Amsterdam University Medical Centre, VUMC, Amsterdam Cardiovascular Sciences Amsterdam The Netherlands; ^6^ Department of Surgery Amsterdam University Medical Centre, VUMC, Amsterdam Cardiovascular Sciences Amsterdam The Netherlands; ^7^ Department of Pathology and Cardiac Surgery Amsterdam University Medical Centre, VUMC, Amsterdam Cardiovascular Sciences Amsterdam The Netherlands; ^8^ Department of Cell and Chemical Biology Leiden University Medical Centre Leiden The Netherlands; ^9^ Department of Clinical Genetics Leiden University Medical Centre Leiden The Netherlands

**Keywords:** Loeys–Dietz syndrome, Myogenic transdifferentiation of fibroblasts, Smooth muscle‐like cells, *TGFBR1* mutation, Thoracic aortic aneurysm and aortic dissection

## Abstract

**Background:**

Thoracic aortic aneurysms and dissections (TAAD) may have a heritable cause in up to 20% of cases. We aimed to investigate the pathogenic effect of a *TGFBR1* mutation in relation to TAAD.

**Methods:**

Co‐segregation analysis was performed followed by functional investigations, including myogenic transdifferentiation.

**Results:**

The c.1043G>A *TGFBR1* mutation was found in the index patient, in a deceased brother, and in five presymptomatic family members. Evidence for pathogenicity was found by the predicted damaging effect of this mutation and the co‐segregation in the family. Functional analysis with myogenic transdifferentiation of dermal fibroblasts to smooth muscle‐like cells, revealed increased myogenic differentiation in patient cells with the *TGFBR1* mutation, shown by a higher expression of myogenic markers *ACTA2*, *MYH11* and *CNN1* compared to cells from healthy controls.

**Conclusion:**

Our findings confirm the pathogenic effect of the *TGFBR1* mutation in causing TAAD in Loeys–Dietz syndrome and show increased myogenic differentiation of patient fibroblasts.

## 1 INTRODUCTION

Clinicians caring for patients with thoracic aortic aneurysms and dissections (TAAD) must be aware of the possible genetic cause of this malformation, because of its diagnostic and therapeutic consequences for both the patient and the family. In up to 20% of cases, a heritable connective tissue disease may be present, which might be either syndromic or nonsyndromic (Albornoz et al., 2006; Coady et al., 1999).

The classical example of syndromic TAAD with a single gene mutation is Marfan syndrome (MFS), caused by mutations in *FBN1*, the gene encoding for fibrillin‐1, an extracellular matrix protein (Cook, Carta, Galatioto, & Ramirez, 2015; De Paepe, Devereux, Dietz, Hennekam, & Pyeritz, 1996; Erbel et al., 2014; Loeys et al., 2010). Another syndromic TAAD is the Loeys–Dietz syndrome (LDS) caused by mutations in transforming growth factor‐β receptor genes *TGFBR1* (OMIM 190181) and *TGFBR2*. LDS was characterized initially by the triad of arterial tortuosity and aneurysms, hypertelorism, craniosynostosis and bifid uvula or cleft palate, including childhood onset aneurysms and dissections (Cozijnsen et al., 2011; Loeys et al., 2005, 2006). Mutations in *TGFBR1* and *TGFBR2* may also cause nonsyndromic familial TAAD (Tran‐Fadulu et al., 2009). Recently a new nosology for LDS was proposed based on genotype only and included also mutations in other genes encoding components of the canonical TGF‐β signaling: LDS1—*TGFBR1* mutation, LDS2—*TGFBR2* mutation, LDS3—*SMAD3* mutation, LDS4—*TGB2* mutation (MacCarrick et al., 2014); more recently LDS5 was added with mutations in *SMAD2* and *TGFB3* (Schepers et al., 2018).

Nonsyndromic familial TAAD may also be caused by mutations in genes of the contractile smooth muscle cell (SMC) apparatus such as *ACTA2* (encoding SMC alpha actin, the thin filament), *MYH11* (encoding SMC myosin heavy chain), or *MYLK*, (encoding myosin light chain kinase).

Mutations in all these different genes cause similar aortic phenotypes (Cook et al., 2015). A recent hypothesis is that altered “mechanosensing” through the elastin‐contractile unit is the major driver of TAAD (Humphrey, Milewicz, Tellides, & Schwartz, 2014; Humphrey, Schwartz, Tellides, & Milewicz, 2015; Milewicz et al., 2017). This was also suggested as a result of mutations in genes encoding components of the canonical TGF‐β signaling (Karimi & Milewicz, 2016). TGF‐β stimulation is a driver of SMC differentiation and this differentiation is defined by increased levels of the contractile proteins, including SMC alpha actin and myosin heavy chain (Guo & Chen, 2012; Yeung et al., 2017). Inamoto et al. showed that aortic SMCs from patients with ascending aortic aneurysms and *TGFBR2* mutations have decreased contractile protein expression including *ACTA2*, *CNN1* and *MYH11*. Furthermore, these SMCs failed to increase expression of these contractile proteins with exposure of TGF‐β1 (Inamoto et al., 2010). For mutations in the *TGFBR1* receptor, SMC differentiation has not been investigated.

Myogenic transdifferentiation is a novel method to generate SMC‐like cells directly from human dermal fibroblasts without genetic manipulation which in itself may affect the differentiation potential of these cells. This method was recently described and shown to be efficient in investigating the pathogenicity of *ACTA2* and *MYH11* variants (Yeung et al., 2017). According to these findings, the properties of the transdifferentiated cells are very comparable to those of primary human SMC with regard to expression of SMC markers and cell contraction.

Our aim was to shed light into the pathogenic effects of the c.1043G>A *TGFBR1* mutation in relation to TAAD. After co‐segregation analysis, functional investigations were performed, including myogenic transdifferentiation. Given the close functional role of TGFBR1 and TGFBR2, we hypothesized that the *TGFBR1* mutation might also dysregulate the function of SMCs as was shown with *TGFBR2* mutations.

## 2 PATIENTS AND METHODS

### 2.1 Patient material and ethical compliance

Clinical data were collected from the Marfan outpatient clinic at the Academic Medical Centre, Amsterdam and the cardiology department at the Gelre Hospital, Apeldoorn. Functional investigations were performed at the department of clinical genetics at the VU University Medical Centre, Amsterdam. For investigation of aortic tissue, signed informed consent was obtained. The study was approved by the Medical Ethical Committee of the VU Medical Centre, Amsterdam. They determined this observational study to fall outside the scope of the Dutch law of medical‐scientific research with humans (WMO).

### 2.2 Clinical and genetic Investigation

The initial clinical and imaging examinations of the relatives were performed within 2.5 years, except for II.3 and the deceased III.1 who had their cardiovascular examination 3 and 15 years earlier, respectively. Diameter measurement of sinus of Valsalva and tubular ascending aorta were performed by echocardiography (leading edge to leading edge, in end‐diastole), eventually supplemented by contrast‐enhanced magnetic resonance imaging (MRI) or computer tomography and angiography (CT). Diameter measurements and comorbidities were retrieved from the electronic medical record. Clinical evaluation (including cardiovascular examination) was performed in 17 family members. Genomic DNA was extracted from peripheral blood leucocytes in 18 family members and from fibroblasts in one deceased patient and mutational analysis of the *TGFBR1, TGFBR2 and FBN1* gene was performed.

### 2.3 Functional analysis

Cultures of primary human skin fibroblasts were established from patients and healthy donors by skin biopsies and analyzed after myogenic transdifferentiation to smooth muscle‐like cells as previously described (Yeung et al., 2017). Details of functional analysis including myogenic transdifferentiation, qPCR, western blotting, immunohistochemistry, and collagen typing are online available as supplemental information. Data were analyzed with IBM SPSS Statistics 22 (IBM Statistics v20). Differences between patients and controls were assessed via the Mann–Whitney test. Tests were considered statistically significant when *p* < .05.

## 3 RESULTS

### 3.1 Patients

**Figure 1:**
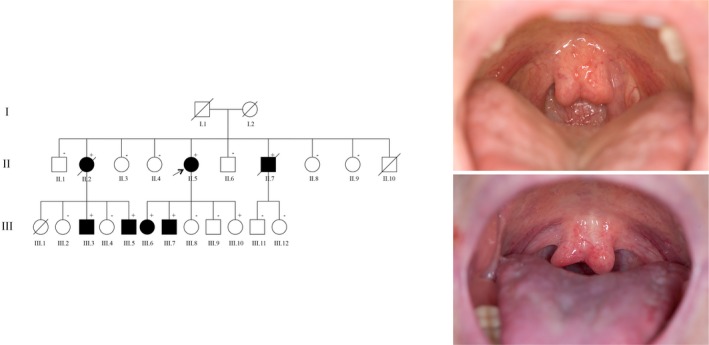
Pedigree of the family. Three generation pedigree of the family. Arrow indicates the index patient II.5. Family members with ± are tested: “+” are carriers (including one deceased, obligate carrier, II.2), “−” are noncarriers. Symbols with slashes indicate deceased family members. Black filled‐in symbols indicate affected mutation carriers. Right: photographs of the mouth of the index patient II.5 and one of her sons III.7 revealing split uvulas

The index patient (II.5, described as an introductory example in a Clinician Update in [Cozijnsen et al., 2011]) was evaluated at the Marfan outpatient clinic for an ascending aortic dilatation (46 mm) at the age of 61 years, for which she underwent supracoronary replacement of the ascending aorta and the proximal aortic arch. At clinical examination, she was found to have a bifid uvula (Figure 1) without other dysmorphic features of LDS. Lumbosacral MRI detected dural ectasia, a common sign in connective tissue disorders. Additional CT scan showed two small cerebral aneurysms of the left internal carotid artery (4 and 5 mm). Four years after aortic surgery, an interposition graft needed to be implanted because of an acute type B aortic dissection. A few months afterwards, she underwent repair of a left axillary artery aneurysm (26 mm). The CT scan showed also dilatations of right subclavian (14 mm), right axillary (16 mm) and right iliac artery (22 mm).

Of her nine sibs (Figure 1), two (II.2 and II.7) had had acute aortic dissections as described in an earlier Case Report (Stroes, Cozijnsen, Jaarsma, Hamerlynck, & Beemer, 1994). The sister (II.2), an obligate carrier, died at the age of 47 years following surgery for type A aortic dissection. Microscopy of the surgical specimen showed medial degeneration with fragmentation of elastic lamellae. Her medical history included mild aortic insufficiency, hypercholesterolemia, and well‐regulated hyperthyroidism. An aneurysm of the right common iliac artery was detected at 41 years of age during abdominal uterus extirpation.

The brother (II.7) underwent a Bentall procedure for type A aortic dissection at the age of 39 years. Histopathological examination revealed extensive medial degeneration with fragmentation of elastic fibers. His medical history included Graves’ thyrotoxicosis and analysis for rheumatic complaints. Four years later, a big thrombus‐filled postdissection aneurysm of the right carotid artery had to be removed and replaced by an interposition graft. At 48 years of age, alopecia areata was diagnosed as well as elbow arthritis with a positive rheumatoid factor test; at 51 years of age, hypothyroidism, and combined hyperlipidemia were established. Because of false aneurysms of the right carotid artery, at the age of 53 years an endovascular stent was implanted; at the age of 56 and 57 years, surgical explorations in the same region needed to be performed. He died at 58 years of age by complications in the vertebrobasilar region of the previously existing dissection.

### 3.2 Genetic analysis

DNA analysis in patient II.5 revealed a mutation in the *TGFBR1* gene (c.1043G>A, p.(Cys348Tyr)) according to the reference sequence NM_004612.2, possibly pathogenic. No mutation was found in the *TGFBR2* gene. Previous DNA‐analysis in patient II.7 had shown no mutation in the *FBN1* gene. Collagen typing in cultured fibroblasts of his skin biopsy was normal (Data S3). The *TGFBR1* mutation was confirmed in fibroblasts of patient II.7. No DNA was available for patient II.2. She was found to be an obligate carrier of the *TGFBR1* mutation. Of the seventeen presymptomatic family members tested, five carried the *TGFBR1* mutation (Figure 1).

The c.1043G>A mutation is currently not reported in databases of human sequence variants, including the NCBI ClinVar database, Single Nucleotide Polymorphism database (dbSNP), Genome Aggregation Database (gnomAD), Exome Sequencing Project (ESP) Variants database, CentoMD Variants database, Human Gene Mutation Database (HGMD Professional), and Genome of the Netherlands (GoNL) Variants. Based on SIFT and Align GVGD programs, it is predicted to have a deleterious effect. The mutation is listed as pathogenic in the database of the Montalcino Aortic Consortium (MAC), an international registry of patients with heritable aortic disease (Jondeau et al., 2016). The p.(Cys348Tyr) mutation results in the substitution of a cysteine with tyrosine in the serine/threonine kinase domain of *TGFBR1*, which is responsible for conveying intracellular signaling. This cysteine is evolutionary and highly conserved from humans down to zebrafish.

### 3.3 Presymptomatic carriers

The five presymptomatic *TGFBR1* mutation carriers ranged in age from 30 to 44 years old. Echocardiography and/or MRI and CT investigations were performed in all. Three (III.3, III.6, III.7) had a dilated ascending aorta (≥40 mm) at initial examination (Table 1). In addition, bifid uvula (Figure 1) and mild tortuosity of arteries was found in one and dural ectasia in one. One family member (III.3) was operated at the age of 6 years because of an aneurysm in the left ventricle of his heart. An infectious cause was suggested, but not confirmed (based on available data). His brother (III.5) had a stroke at the age of 35 years caused by the occlusion of the right posterior cerebral artery. Syndromal signs of LDS were few, but features of connective tissue disorders (dural ectasia, high and/or narrow palate, and pectus deformity) were found more often.

**Table 1:** Details of family members

	Age at investigation (years)	Sinus of Valsalva (mm)	Tubular ascending aorta (mm)	MRI/CT findings	Other vascular anomalies	Physical features	Auto‐immune disease
Carriers							
II.2	47	Type A dissection			+		TH
II.5	61	37	46	DE/CA	+	BU	
II.7	39	Type A dissection		CO			RF, TH
III.3	44	42	33		+		
III.5	35	37	26		+	PA/PD	
III.6	44	44	38	DE		PA	UC
III.7	42	40	38	TO		BU/PD	TH
III.10	31	ND	31				
Noncarriers							
II.1	71	ND	ND				
II.3	62	39	35				SJ, TH
II.4	65	31	27				PMR
II.6	61	36	34				
II.8	59	35	34				CC
II.9	54	32	34				
II.10	3 days^a^						
III.1	34 years^a^, ^b^	38^c^	26^c^				
III.2	46	27	28				
III.4	35	28	28				
III.8	30	ND	28			PA	
III.9	36	29	25				
III.11	33	32	30				
III.12	27	32	28				

Abbreviations: +, Present; BU, bifid uvula; CA, cerebral aneurysm; CC, collagenous colitis; CO, cerebral occlusion; DE, dural ectasia; ND, no data; PA, high/narrow palate; PD, pectus deformity; PMR, polymyalgia rheumatica; RF, Rheumatic complaints with positive rheumatic factor test; SJ, Sjögren's syndrome; TH, thyroid dysfunction; TO, tortuosity; UC, ulcerative colitis.

a Died.

b Ovarian cancer.

c At 31 years of age.

After six years follow‐up, at 49 years of age, one of the mutation carriers (III.2) underwent a prophylactic aortic root replacement (valve sparing) because of progressive root dilatation (46 mm). The aortic root diameter of his brother (III.5) had progressed to 40 mm. The youngest carrier (III.10) still had a nondilated ascending aorta (31 mm) at the age of 37.

### 3.4 Other family members

Among the eleven presymptomatic individuals tested in whom the *TGFBR1* mutation was not found, all had an echocardiography and/or MRI performed. None of them had a dilation of the ascending aorta (all <40 mm).

It was unclear from whom the index patient and her affected sibs had inherited the *TGFBR1* mutation. Their father (I.1) had a stroke at 41 years of age and had rheumatic arthritis. He died 10 years later due to hepatocellular carcinoma. Autopsy was not performed. Their mother (I.2) had a myocardial infarction at 71 years of age and died 12 years later due to cerebral bleeding. There was no clear history of aortic aneurysm/dissection in either family.

### 3.5 Functional analysis

**Figure 2:**
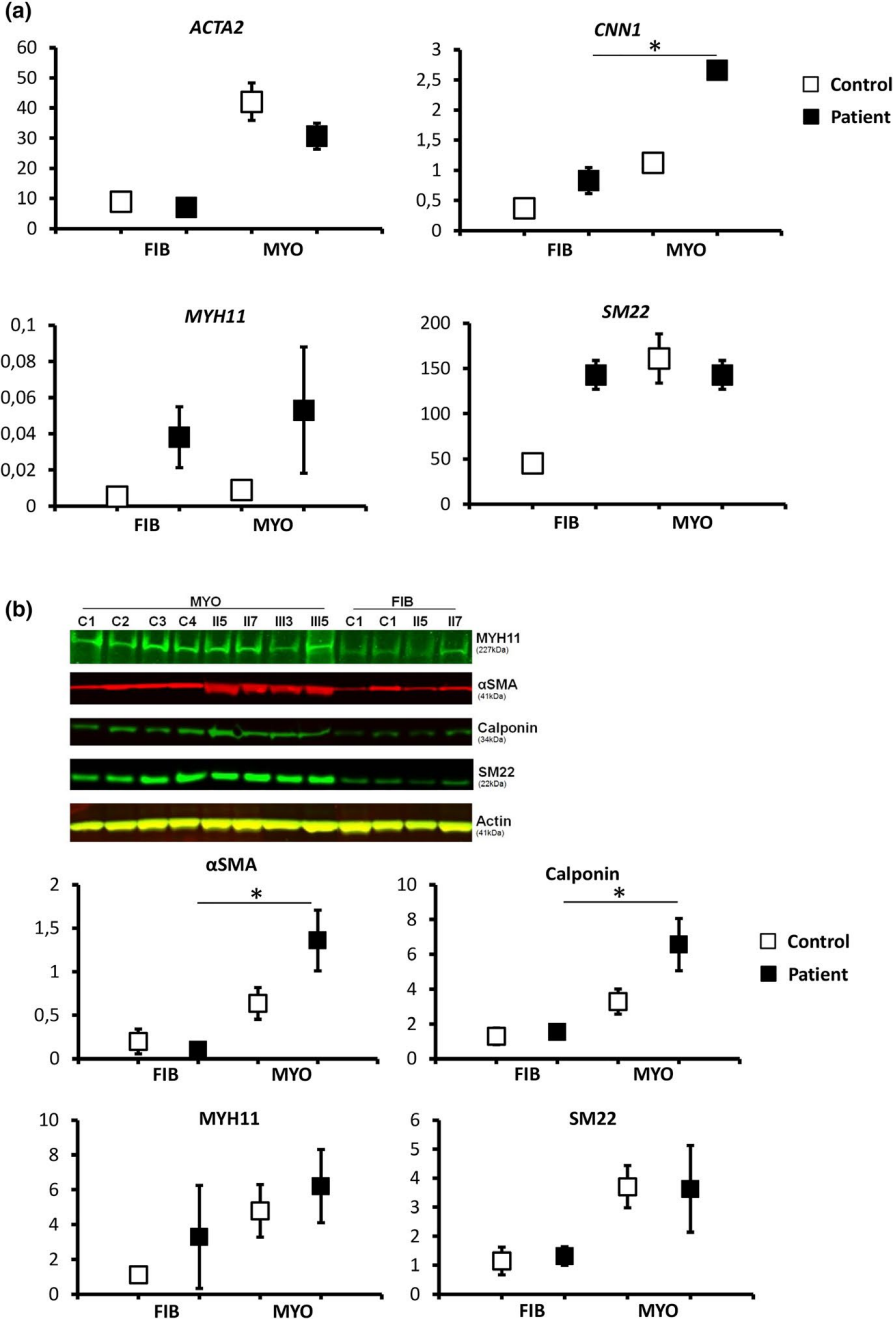
Analysis of myogenic transdifferentiation. (a) Increased myogenic transdifferentiation of fibroblasts of patients with the *TGFBR1* c.1043G>A mutation. Dermal fibroblasts (FIB) from patients (*n* = 4) and healthy controls (*n* = 4) were subjected to myogenic transdifferentiation (MYO) for 14 days. Expression of *ACTA2*, *SM22*, *CNN1* and *MYH11* mRNA was measured by qPCR. Relative expression was normalized based on expression of *TBP*. Each square indicates the average of gene expression per group per condition and error bars ± standard deviation. p values lower than .05 (*p* < .05) were considered to be significant based on Mann–Whitney test. (b) Protein expression of myogenic markers after myogenic transdifferentiation of fibroblasts with the *TGFBR1* c.1043G>A mutation. Dermal fibroblasts (FIB) of the indicated patients and healthy controls (c) were subjected to myogenic transdifferentiation (MYO) for 14 days. Expression of αSMA, calponin, MYH11 and SM22 in whole cell lysates was measured by western blotting. Actin was used as loading control. The expression was quantified and normalized based on the expression of the housekeeping gene. Each square indicates the average of patients or controls, before (FIB) or after myogenic transdifferentiation (MYO); errors bars show the standard deviation per group. Differences were significant (*) when *p* < .05 measured by Mann–Whitney test

In order to investigate the effect of the *TGFBR1* mutation on myogenic potential, we subjected fibroblasts from healthy donors and 4 affected family members (II.5, II.7, III.3 and III.5) with confirmed mutation to myogenic transdifferentiation. This was performed based on our established protocol (Yeung et al., 2017) as previously described which enables the transdifferentiation of fibroblasts to SMC‐like cells as shown by upregulation of the myogenic markers ACTA2, CNN, MYH11 and SM22. In these SMC‐like cells, the average expression of CNN1 (*p* < .05) and MYH11 myogenic markers was higher in patient cells than that in cells from healthy controls at the mRNA level (Figure 2a). The same was observed for αSMA (*p* < .05), Calponin (*p* < .05) and MYH11 on the protein level (Figure 2b).

Given that both activin A and TGF‐β mediate signaling through SMAD2 and SMAD3, we stimulated fibroblasts from patients II.5 and II.7 with activin A or TGF‐β1 to determine if the mutation can affect signaling through the TGFBR1 receptor. Although induction of phosphorylated SMAD3 was observed in the patient cells, no differences in the expression of phosphorylated SMAD3 were observed compared to fibroblasts from healthy donors for both TGF‐β1 and Activin A (Figure S1).

Immunohistochemical analysis revealed upregulation of total and phosphorylated SMAD3 in an aortic biopsy from patient III.3 and patient II.5 obtained by cross section. The staining for total SMAD3 was mostly observed in the nucleus of SMCs. The results were compared to biopsies from patients with atherosclerosis in which no staining for total SMAD3 was observed (Figure S2). Given that SMAD3 activation by phosphorylation is required for the translocation of SMAD3 to the nucleus, this indicates that SMAD3 is mostly found in its phosphorylated form in the SMCs of the media section. This was confirmed by immunohistochemistry analysis for phosphorylated SMAD3; SMCs in the media section of the patient tested positive for phosphorylated SMAD3 in the nucleus, whereas little staining was found in SMCs of the controls.

## 4 DISCUSSION

### 4.1 Main results

In a patient with TAAD and bifid uvula, we discovered a mutation in the *TGFBR1* gene (c.1043G>A, p. (Cys348Tyr)) that caused type A aortic dissections in two family members and ascending aortic dilatations in another five family members, whereby LDS was diagnosed. Evidence for the pathogenicity of the mutation was as follows: (a) the segregation with the disease in the family, (b) the predicted inactivating effect of this mutation in the highly conserved kinase domain which has a critical role in intracellular signal transduction, and (c) the listing as pathogenic in the MAC database.

Additionally, we were the first to investigate myogenic transdifferentiation in fibroblasts of patients with the *TGFBR1* mutation by which we observed increased myogenic differentiation in four patients as shown by a higher expression of myogenic markers compared to cells from healthy controls.

The mutation was not found to affect the mRNA expression of *TGFBR1* compared to healthy volunteer cells (data not shown). *TGFBR1* null mutations cause multiple self‐healing squamous epithelioma (MSSE) (Goudie et al., 2011), while deletion of *TGFBR1* in mice promotes squamous carcinoma (Bian et al., 2009). The phenotypic difference indicates that the effect of this *TGFBR1* mutation potentially extends beyond the loss of function to a dominant negative effect.

Despite the damaging effect of the *TGFBR1* c.1043G>A mutation on the kinase domain of this receptor, we detected increased pSMAD3 expression which indicates increased SMAD signaling in SMCs in the media section of the aorta. This was in line with previous studies which report a “paradoxical” activation of SMAD signaling following inactivating mutations in components of TGF‐β signaling as a fundamental mechanism underlying thoracic aneurysm pathology (Akhurst, 2012; Loeys et al., 2005). This activation was not detected in fibroblasts of the patients in agreement with a previous study (Loeys et al., 2005). This is as expected, given that dermal fibroblasts are not directly the relevant cell type for the study of aortic pathology. Despite the fact that LDS patients can show cutaneous features no reports exist about aberrant SMAD signaling in dermal fibroblasts of LDS patients as a result of *TGFBR1* or *TGFBR2* mutations. However, fibroblasts can serve as an obtainable cell type that can be easily differentiated to SMC‐like cells, the shown affected cell type in aortic disease in LDS.

The amount of autoimmune diseases in the family was remarkable, but appeared not to be associated with the *TGFBR1* mutation, nor with a gene close to chromosome 9q22. This is in agreement with previous studies reporting immune dysregulation in LDS patients (Felgentreff et al., 2014; Guerrerio et al., 2016).

### 4.2 Increased myogenic differentiation

We also report increased myogenic differentiation in fibroblasts of patients with the *TGFBR1* c.1043G>A mutation as shown by the significant upregulation of SMC‐specific proteins, mainly αSMA and calponin. This is in contrast with the results of Inamoto et al. who observed diminished expression of ACTA2, CNN1 and MYH11. Their observations were made in explanted ascending aortic SMCs from nonrelated familial TAAD or LDS patients with a *TGFBR2* mutation, as opposed to ours in transdifferentiated SMC‐like cells of four related patients with the *TGFBR1* mutation.(Inamoto et al., 2010) Nonetheless, our results are in line with the findings of Crosas‐Molist et al. showing increased expression of contractile protein markers in explanted SMCs from dilated ascending aortas of MFS patients in comparison with healthy controls. The latter study claims the overexpression of contractile proteins to be largely associated with the overactivation of TGF‐β signaling which is implicated in arterial stiffness leading to aneurysm formation (Crosas‐Molist et al., 2015). Moreover, premature differentiation of SMCs has been shown to play a role in aortic aneurysms of a murine MFS model (Dale et al., 2017). Given that excessive SMAD signaling is the common denominator in all syndromic and nonsyndromic aneurysms (Gomez et al., 2009) and that TGF‐β has a key function as driver of SMC differentiation (Guo & Chen, 2012), our findings suggest that increased SMC differentiation might be a possible underlying driver toward aneurysm development in patients with this pathogenic *TGFBR1* mutation. However, we have not been able to examine SMAD signaling in the SMC‐like cells due to low cell numbers used for the transdifferentiation. Further investigation is required to confirm this observation in aortic SMCs of TAAD patients with other *TGFBR1* mutations and with mutations in other aneurysm‐related genes.

A limitation of the functional study is the small number of samples, limited to the four related patients with the same mutation. Another limitation is the variation in the transdifferentiation potential between cell lines despite the same genetic defect in *TGFBR1*. This limitation of this method can be possibly due to the heterogeneity of dermal fibroblast populations (Sorrell & Caplan, 2004). It will be meaningful in future studies to confirm our findings on myogenic transdifferentiation in primary SMCs of the patients which is, however, not always possible owing to the invasiveness of aortic biopsies.

In conclusion, our findings confirm the c.1043G>A *TGFBR1* mutation as the cause of Loeys–Dietz syndrome. This mutation may exert its pathogenic effect by perturbation of myogenic differentiation, evidence for which has to be provided in future investigations of the aorta pathology.

## CONFLICT OF INTEREST

None of the authors have any conflicts of interest to report.

## References

[mgg3943-bib-0001] Akhurst, R. J. (2012). The paradoxical TGF‐beta vasculopathies. Nature Genetics, 44, 838–839. 10.1038/ng.2366 22836090 PMC3543110

[mgg3943-bib-0002] Albornoz, G. , Coady, M. A. , Roberts, M. , Davies, R. R. , Tranquilli, M. , Rizzo, J. A. , & Elefteriades, J. A. (2006). Familial thoracic aortic aneurysms and dissections–incidence, modes of inheritance, and phenotypic patterns. Annals of Thoracic Surgery, 82, 1400–1405. 10.1016/j.athoracsur.2006.04.098 16996941

[mgg3943-bib-0003] Bian, Y. , Terse, A. , Du, J. , Hall, B. , Molinolo, A. , Zhang, P. , et al. (2009). Progressive tumor formation in mice with conditional deletion of TGF‐beta signaling in head and neck epithelia is associated with activation of the PI3K/Akt pathway. Cancer Research, 69, 5918–5926. 10.1158/0008-5472.CAN-08-4623 19584284 PMC2758611

[mgg3943-bib-0004] Coady, M. A. , Davies, R. R. , Roberts, M. , Goldstein, L. J. , Rogalski, M. J. , Rizzo, J. A. , et al. (1999). Familial patterns of thoracic aortic aneurysms. Archives of Surgery, 134, 361–367. 10.1001/archsurg.134.4.361 10199307

[mgg3943-bib-0005] Cook, J. R. , Carta, L. , Galatioto, J. , & Ramirez, F. (2015). Cardiovascular manifestations in Marfan syndrome and related diseases; multiple genes causing similar phenotypes. Clinical Genetics, 87, 11–20. 10.1111/cge.12436 24867163

[mgg3943-bib-0006] Cozijnsen, L. , Braam, R. L. , Waalewijn, R. A. , Schepens, M. A. A. M. , Loeys, B. L. , van Oosterhout, M. F. M. , … Mulder, B. J. M. (2011). What is new in dilatation of the ascending aorta? Review of current literature and practical advice for the cardiologist. Circulation, 123, 924–928. 10.1161/CIRCULATIONAHA.110.949131 21357847

[mgg3943-bib-0007] Crosas‐Molist, E. , Meirelles, T. , López‐Luque, J. , Serra‐Peinado, C. , Selva, J. , Caja, L. , … Egea, G. (2015). Vascular smooth muscle cell phenotypic changes in patients with Marfan syndrome. Arteriosclerosis, Thrombosis, and Vascular Biology, 35, 960–972. 10.1161/ATVBAHA.114.304412 25593132

[mgg3943-bib-0008] Dale, M. , Fitzgerald, M. P. , Liu, Z. , Meisinger, T. , Karpisek, A. , Purcell, L. N. , et al. (2017). Premature aortic smooth muscle cell differentiation contributes to matrix dysregulation in Marfan Syndrome. PLoS ONE, 12, e0186603. 29040313 10.1371/journal.pone.0186603 PMC5645122

[mgg3943-bib-0009] De Paepe, A. , Devereux, R. B. , Dietz, H. C. , Hennekam, R. C. , & Pyeritz, R. E. (1996). Revised diagnostic criteria for the Marfan syndrome. American Journal of Medical Genetics, 62, 417–426. 10.1002/(SICI)1096-8628(19960424)62:4<417:AID-AJMG15>3.0.CO;2-R 8723076

[mgg3943-bib-0010] Erbel, R. , Aboyans, V. , Boileau, C. , Bossone, E. , Di, B. R. , Eggebrecht, H. , et al. (2014). 2014 ESC Guidelines on the diagnosis and treatment of aortic diseases: Document covering acute and chronic aortic diseases of the thoracic and abdominal aorta of the adultThe Task Force for the Diagnosis and Treatment of Aortic Diseases of the European Society of Cardiology (ESC). European Heart Journal, 35, 2873–2926. 10.1093/eurheartj/ehu281 25173340

[mgg3943-bib-0011] Felgentreff, K. , Siepe, M. , Kotthoff, S. , von Kodolitsch, Y. , Schachtrup, K. , Notarangelo, L. D. , … Ehl, S. (2014). Severe eczema and Hyper‐IgE in Loeys‐Dietz‐syndrome ‐ contribution to new findings of immune dysregulation in connective tissue disorders. Clinical Immunology, 150, 43–50. 10.1016/j.clim.2013.11.008 24333532

[mgg3943-bib-0012] Gomez, D. , Al Haj Zen, A. , Borges, L. F. , Philippe, M. , Gutierrez, P. S. , Jondeau, G. , … Vranckx, R. (2009). Syndromic and non‐syndromic aneurysms of the human ascending aorta share activation of the Smad2 pathway. The Journal of Pathology, 218, 131–142. 10.1002/path.2516 19224541

[mgg3943-bib-0013] Goudie, D. R. , D'Alessandro, M. , Merriman, B. , Lee, H. , Szeverényi, I. , Avery, S. , … Lane, E. B. (2011). Multiple self‐healing squamous epithelioma is caused by a disease‐specific spectrum of mutations in TGFBR1. Nature Genetics, 43, 365–369. 10.1038/ng.780 21358634

[mgg3943-bib-0014] Guerrerio, A. L. , Frischmeyer‐Guerrerio, P. A. , Huang, C. , Wu, Y. , Haritunians, T. , McGovern, D. P. B. , … Dietz, H. C. (2016). Increased prevalence of inflammatory bowel disease in patients with mutations in genes encoding the receptor subunits for TGFβ. Inflammatory Bowel Diseases, 22, 2058–2062. 10.1097/MIB.0000000000000872 27508510 PMC4992461

[mgg3943-bib-0015] Guo, X. , & Chen, S. Y. (2012). Transforming growth factor‐beta and smooth muscle differentiation. World Journal of Biological Chemistry, 3, 41–52. 10.4331/wjbc.v3.i3.41 22451850 PMC3312200

[mgg3943-bib-0016] Humphrey, J. D. , Milewicz, D. M. , Tellides, G. , & Schwartz, M. A. (2014). Cell biology. Dysfunctional Mechanosensing in Aneurysms. Science, 344, 477–479. 10.1126/science.1253026 24786066 PMC4360903

[mgg3943-bib-0017] Humphrey, J. D. , Schwartz, M. A. , Tellides, G. , & Milewicz, D. M. (2015). Role of mechanotransduction in vascular biology: Focus on thoracic aortic aneurysms and dissections. Circulation Research, 116, 1448–1461. 10.1161/CIRCRESAHA.114.304936 25858068 PMC4420625

[mgg3943-bib-0018] Inamoto, S. , Kwartler, C. S. , Lafont, A. L. , Liang, Y. Y. , Fadulu, V. T. , Duraisamy, S. , … Milewicz, D. M. (2010). TGFBR2 mutations alter smooth muscle cell phenotype and predispose to thoracic aortic aneurysms and dissections. Cardiovascular Research, 88, 520–529. 10.1093/cvr/cvq230 20628007 PMC2972687

[mgg3943-bib-0019] Jondeau, G. , Ropers, J. , Regalado, E. , Braverman, A. , Evangelista, A. , Teixedo, G. , et al. (2016). International registry of patients carrying TGFBR1 or TGFBR2 mutations: Results of the MAC (Montalcino Aortic Consortium). Circulation: Cardiovascular Genetics, 9, 548–558. 10.1161/CIRCGENETICS.116.001485 27879313 PMC5177493

[mgg3943-bib-0020] Karimi, A. , & Milewicz, D. M. (2016). Structure of the elastin‐contractile units in the thoracic aorta and how genes that cause thoracic aortic aneurysms and dissections disrupt this structure. Canadian Journal of Cardiology, 32, 26–34. 10.1016/j.cjca.2015.11.004 26724508 PMC4839280

[mgg3943-bib-0022] Loeys, B. L. , Chen, J. , Neptune, E. R. , Judge, D. P. , Podowski, M. , Holm, T. , … Dietz, H. C. (2005). A syndrome of altered cardiovascular, craniofacial, neurocognitive and skeletal development caused by mutations in TGFBR1 or TGFBR2. Nature Genetics, 37, 275–281. 10.1038/ng1511 15731757

[mgg3943-bib-0023] Loeys, B. L. , Dietz, H. C. , Braverman, A. C. , Callewaert, B. L. , De Backer, J. , Devereux, R. B. , … De Paepe, A. M. (2010). The revised Ghent nosology for the Marfan syndrome. Journal of Medical Genetics, 47, 476–485. 10.1136/jmg.2009.072785 20591885

[mgg3943-bib-0024] Loeys, B. L. , Schwarze, U. , Holm, T. , Callewaert, B. L. , Thomas, G. H. , Pannu, H. , et al. (2006). Aneurysm syndromes caused by mutations in the TGF‐beta receptor. The New England Journal of Medicine, 355, 788–798. 10.1056/NEJMoa055695 16928994

[mgg3943-bib-0025] MacCarrick, G. , Black, J. H. , Bowdin, S. , El‐Hamamsy, I. , Frischmeyer‐Guerrerio, P. A. , Guerrerio, A. L. , … Dietz, H. C. (2014). Loeys‐Dietz syndrome: A primer for diagnosis and management. Genetics in Medicine, 16, 576–587. 10.1038/gim.2014.11 24577266 PMC4131122

[mgg3943-bib-0026] Milewicz, D. M. , Trybus, K. M. , Guo, D.‐C. , Sweeney, H. L. , Regalado, E. , Kamm, K. , & Stull, J. T. (2017). Altered smooth muscle cell force generation as a driver of thoracic aortic aneurysms and dissections. Arteriosclerosis, Thrombosis, and Vascular Biology, 37, 26–34. 10.1161/ATVBAHA.116.303229 PMC5222685 27879251

[mgg3943-bib-0027] Schepers, D. , Tortora, G. , Morisaki, H. , MacCarrick, G. , Lindsay, M. , Liang, D. , et al. (2018). A mutation update on the LDS‐associated genes TGFB2/3 and SMAD2/3. Human Mutation, 39, 621–634. 10.1002/humu.23407 29392890 PMC5947146

[mgg3943-bib-0028] Sorrell, J. M. , & Caplan, A. I. (2004). Fibroblast heterogeneity: More than skin deep. Journal of Cell Science, 117, 667–675. 10.1242/jcs.01005 14754903

[mgg3943-bib-0029] Stroes, E. S. C. , Cozijnsen, L. , Jaarsma, W. , Hamerlynck, R. P. H. M. , & Beemer, F. A. (1994). Familiale acute aortadissectie. Cardiologie, 68–71.

[mgg3943-bib-0030] Tran‐Fadulu, V. , Pannu, H. , Kim, D. H. , Vick, G. W. , Lonsford, C. M. , Lafont, A. L. , … Milewicz, D. M. (2009). Analysis of multigenerational families with thoracic aortic aneurysms and dissections due to TGFBR1 or TGFBR2 mutations. Journal of Medical Genetics, 46, 607–613. 10.1136/jmg.2008.062844 19542084

[mgg3943-bib-0031] Yeung, K. K. , Bogunovic, N. , Keekstra, N. , Beunders, A. A. , Pals, J. , van der Kuij, K. , et al. (2017). Transdifferentiation of human dermal fibroblasts to smooth muscle‐like cells to study the effect of MYH11 and ACTA2 mutations in aortic aneurysms. Human Mutation, 38, 439–450. 10.1002/humu.23174 28074631

