# Supplementary figures and images for: Pathogenic effect of a TGFBR1 mutation in a family with Loeys–Dietz syndrome
Source: Mol Genet Genomic Med. 2019 Sep 1;7(10):e00943. doi: 10.1002/mgg3.943 (PMC6785444; doi:10.1002/mgg3.943)

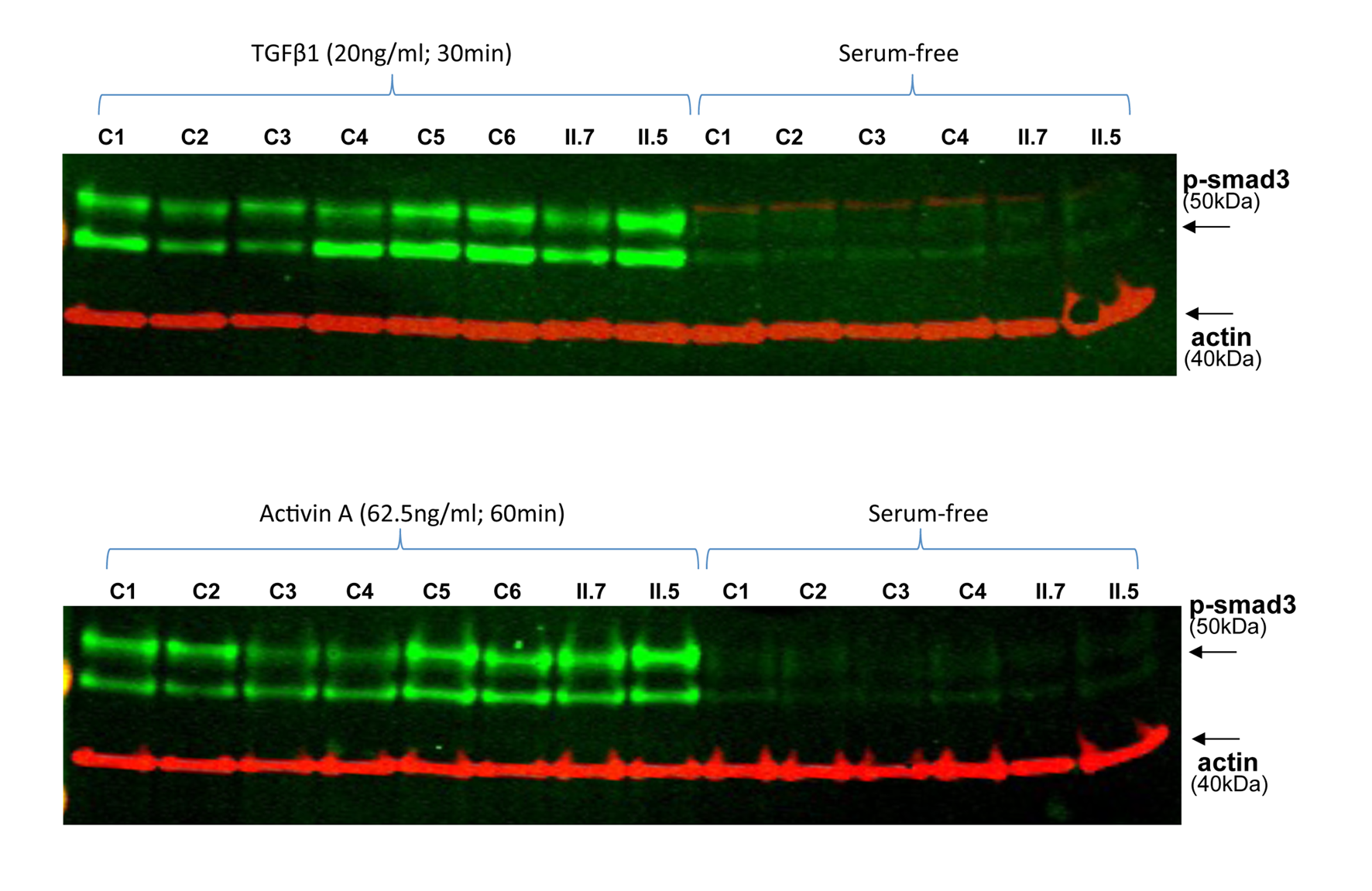

Supplement: Supplementary file 1 [file MGG3-7-e00943-s001.tif]

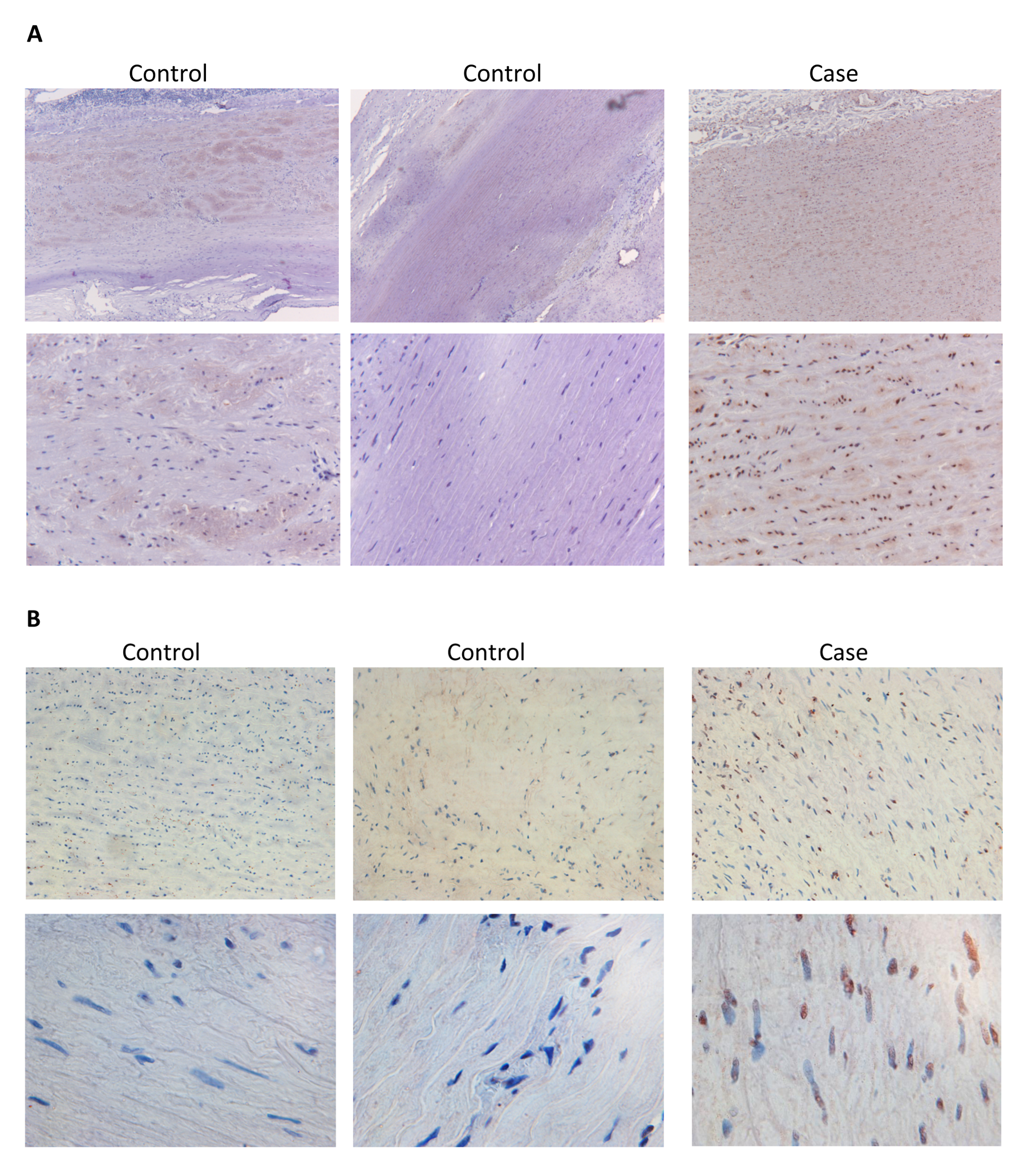

Supplement: Supplementary file 2 [file MGG3-7-e00943-s002.tif]
