# Supplementary material for: Pathogenic effect of a TGFBR1 mutation in a family with Loeys–Dietz syndrome
Source: Mol Genet Genomic Med. 2019 Sep 1;7(10):e00943. doi: 10.1002/mgg3.943 (PMC6785444; doi:10.1002/mgg3.943)
Supplement: Supplementary file 3 [file MGG3-7-e00943-s003.docx]

**Supplementary material** (online publication only)

**SUPPLEMENTARY TEXT**

**S1. Functional analysis**

Cultures of primary human skin fibroblasts were established from patients and healthy donors by skin biopsies. Skin biopsies were performed with a skin punch. The skin biopsy was left to adhere overnight on T25 cell culture flasks, next day 2ml of Ham’s F10 Nutrient Mix media was added, supplemented with 10% FCS and 1% penicillin/streptomycin (Thermo Fisher Scientific). In a period of 2 weeks, fibroblasts migrated from the biopsy and a culture of cells was established. Cells were grown in a humidified condition at 37°C and 5% CO_2_.

Myogenic transdifferentiation of primary skin fibroblasts was performed as previously described (Yeung et al., 2017). Skin fibroblasts were seeded on matriderm (MedSkin Solutions) at 250,000/cm^2^ and they were treated for 14 days with myogenic media consisting of DMEM with 2% heat-inactivated equine serum (Thermo Fisher Scientific) and 1 or 5ng/ml TGF-β1 (BioVision Inc).

Quantitative polymerase chain reaction (qPCR) was performed by the following steps. Fibroblasts subjected to myogenic transdifferentiation were used for mRNA isolation which was performed with the NucleoSpin TriPrep kit (Macherey-Nagel) according to manufacturer’s instructions. 20ng of isolated RNA was used to synthesize cDNA with the VILO kit (Thermo Fisher Scientific). After diluting 5x the cDNA, qPCR was carried out with SYBR Green qPCR mastermix (Roche Diagnostics) at an end concentration of 0.1pmol/µl for each of the reverse and forward primers. The qPCR program consisted of 45 cycles of amplification, and each cycle was performed at 95°C for 10sec, 60°C for 5sec, and 72°C for 10sec. The Lightcycler PCR platform equipped with the Light Cycler 480 release 1.5.0 SP4 software (Roche) was used to perform the qPCR reactions and analyze the data. qPCR was performed for the myogenic markers *ACTA2*, *SM22*, *CNN1,* and *MYH11*; the expression of the housekeeping gene *TBP* was used to normalize results based on the cell number. The respective primer sets are shown in Supplemental Table S1.

Western blotting was performed as follows. Whole cell lysates of cultured fibroblasts were prepared by lysing in NuPAGE 4x sample buffer with reducing agent. Cells lysates were used for electrophoresis in Bis-Tris NuPAGE gels in MOPS running buffer. Proteins were transferred to nitrocellulose using the iBLOT transfer system (Invitrogen). The nitrocellulose membrane was incubated in blocking buffer for 1h (LICOR) before being probed overnight with antibodies against phosphorylated SMAD3 (abcam, Cat#ab52903), SM22(abcam, Cat#ab155272), αSMA (Dako, Cat#M0851), MYH11 (abcam, Cat#ab53219) and actin (abcam, Cat#ab14128). Secondary antibody incubation was conducted with IRDye-conjugated antibodies, goat anti-rabbit and goat anti-mouse respectively (LICOR Biosciences). Fluorescence was visualized with the Odyssey version 4 software of the Odyssey scanner (LICOR Biosciences) which was also used for the quantification of protein expression by measuring the integrated intensity of pixels in the selected area for the investigated and housekeeping proteins.

**S2. Immunohistochemistry**

For immunohistochemical analysis, formalin-fixed paraffin embedded tissue was cut into 4µm sections, deparaffinised, rehydrated and incubated in methanol/H_2_O_2_ (0.3%) for 30min to block endogenous peroxidases. Antigen retrieval was performed by boiling in 10mM Tris/1mM EDTA-buffer, pH 9.0. The slides were incubated with either rabbit antihuman totSMAD3 (1:200; Abcam), or rabbit antihuman pSMAD3 (1:100, Abcam) for 1h at room temperature. The slides with rabbit antihuman pSMAD3 (1/100 Abcam) were incubated overnight at room temperature. The sections were then washed with phosphate-buffered saline and incubated with EnVision HRP α-rabbit (undiluted; Dako) for 30min at room temperature. The totalSMAD3 stainings were visualized using 3,3′-diaminobenzidine (DAB; 0.1mg/ml, 0.02% H_2_O_2_). The pSMAD3 stainings were visualized using aminoethyl carbazole (AEC Single solution, Invitrogen). The sections were then counterstained with haematoxylin, dehydrated, and covered. For each staining, a phosphate-buffered saline control was included which yielded negative results (data not shown). Specimens were visualised with the Leica DM4000B microscope which is coupled to the Leica MC170HD camera. Photos were made with the Leica microsystems LAS v4.12 software.

**S3. Collagen typing**

Collagen typing in cultured fibroblasts from skin biopsy was determined by electrophoresis of ^14^C proline-labeled collagen as previously described after small adaptation of the protocol (Korkko et al., 1997). Briefly, ^14^C proline solution (12.5µCi) was added to confluent cells in combination with vitamin C stimulation. Collagen in cell lysates and cell culture media was solubilised in 0.5M acetic acid followed by treatment with pepsin to convert procollagen to collagen. Samples were then freeze-dried and subjected to electrophoresis 11.5% acryl/bisacryl-urea-polyacrylamide gels at 200V and ~110mA for 90min after which 7.7mg/ml DTT in sucrose was added per well to facilitate the entering of collagen type III α chains in the gel. Electrophoresis was then continued at 400V and ~95A for 90min. Finally, gels were vacuum-dried and quantified by phosphorimaging.

**SUPPLEMENTARY TABLES**

**TABLE S1** Primer sequence list

| **Gene name** | **Accession number** | **Primers** |
| --- | --- | --- |
| *ACTA2* | [NM_001141945](http://www.ncbi.nlm.nih.gov/nuccore/NM_001141945) | ACTGGGACGACATGGAAAAG |
|  |  | CATACATGGCTGGGACATTG |
| *CNN1* | [NM_001308341](http://www.ncbi.nlm.nih.gov/nuccore/NM_001308341) | GCCCAGAAGTATGACCACCA |
|  |  | TGATGAAGTTGCCGATGTTC |
| *SM22* | NM_001001522 | AAGAATGATGGGCACTACCG |
|  |  | AGCCCTCTCCGCTCTAACTG |
| *MYH11* | [NM_001040113](http://www.ncbi.nlm.nih.gov/nuccore/NM_001040113) | CAGCCAGCATTAAGGAGGAG |
|  |  | GCAGAAGAGGCCAGAGTACG |
| *TBP* | [NM_003194](http://www.ncbi.nlm.nih.gov/nuccore/NM_003194) | AGTTCTGGGATTGTACCGCA |
|  |  | TCCTCATGATTACCGCAGCA |

**TABLE S2** Characteristics of donor cell lines

| **Donor** | **Gender** | **Age at biopsy (years)** |
| --- | --- | --- |
| **Control 1** | **Male** | **44** |
| **Control 2** | [**Female**](http://www.ncbi.nlm.nih.gov/nuccore/NM_001308341) |  |
|  |  | **54** |
| **Control 3** | **Female** | **40** |
| **Control 4** | [**Female**](http://www.ncbi.nlm.nih.gov/nuccore/NM_001040113) | **48** |
| **Control 5** | [**Male**](http://www.ncbi.nlm.nih.gov/nuccore/NM_001040113) | **58** |
| **Control 6** | [**Female**](http://www.ncbi.nlm.nih.gov/nuccore/NM_001040113) | **52** |
| **Patient II.5** | **Female** | **64** |
| **Patient II.7** | **Male** | **42** |
| **Patient III.3** | **Male** | **53** |
| **Patient III.5** | **Male** | **43** |

**SUPPLEMENTARY FIGURE LEGENDS**

**FIGURE S1** Expression of phosphorylated SMAD3 in dermal fibroblasts

Expression of phosphorylated SMAD3 after stimulation with activin A or TGF-β1 in dermal fibroblasts of 2 patients (II.5 and II.7) and 6 healthy controls. After overnight FCS starvation cells were stimulated with TGF-β1 and activin A as indicated. The expression of phosphorylated SMAD3 was measured by western blotting. Actin was used to normalize for equal protein loading.

**FIGURE S2** Immunohistochemistry of SMAD3 in aortic tissue

Immunohistochemistry of total SMAD3 and phosphorylated SMAD3 in the aortic wall of patients with the *TGFBR1* c.1043G>A mutation compared to atherosclerotic controls. Images show cross-section of the aortic tissue mostly depicting the media layer with SMCs. Increased total SMAD3 staining is observed in SMCs of patient III.3 compared to controls; upper and lower rows show 5x and 20x magnification respectively (A). In patient II.5 staining for phosphorylated SMAD3 is shown in the nucleus of SMCs; upper and lower rows show 10x and 40x magnification respectively (B).
